# Resveratrol, an Nrf2 activator, ameliorates aging-related progressive renal injury

**DOI:** 10.18632/aging.101361

**Published:** 2018-01-11

**Authors:** Eun Nim Kim, Ji Hee Lim, Min Young Kim, Tae Hyun Ban, In-Ae Jang, Hye Eun Yoon, Cheol Whee Park, Yoon Sik Chang, Bum Soon Choi

**Affiliations:** 1 Division of Nephrology, Department of Internal Medicine, College of Medicine, The Catholic University of Korea, Seoul, Republic of Korea; 2 Department of Internal Medicine, Seoul St. Mary’s Hospital, College of Medicine, The Catholic University of Korea, Seoul, Republic of Korea; 3 Department of Internal Medicine, Incheon St. Mary’s Hospital, College of Medicine, The Catholic University of Korea, Seoul, Republic of Korea; 4 Department of Internal Medicine, Yeouido St. Mary’s Hospital, College of Medicine, The Catholic University of Korea, Seoul, Republic of Korea

**Keywords:** aging, kidney, resveratrol, SIRT1-Nrf2, oxidative stress

## Abstract

Background. Two important issues in the aging kidney are mitochondrial dysfunction and oxidative stress. An Nrf2 activator, resveratrol, is known to have various effects. Resveratrol may prevent inflammation and oxidative stress by activating Nrf2 and SIRT1 signaling. We examined whether resveratrol could potentially ameliorate the cellular condition, such as renal injury due to cellular oxidative stress and mitochondrial dysfunction caused by aging.

Methods. Male 18-month-old C57BL/6 mice were used. Resveratrol (40 mg/kg) was administered to aged mice for 6 months. We compared histological changes, oxidative stress, and aging-related protein expression in the kidney between the resveratrol-treated group (RSV) and the control group (cont). We performed experiments using small-interfering RNAs (siRNAs) for Nrf2 and SIRT1 in cultured HK2 cells.

Results. Resveratrol improved renal function, proteinuria, histological changes and inflammation in aging mice. Also, expression of Nrf2-HO-1-NOQ-1 signaling and SIRT1-AMPK-PGC-1α signaling was increased in the RSV group. Transfection with Nrf2 and SIRT1 siRNA prevented resveratrol-induced anti-oxidative effect in HK2 cells in media treated with H_2_O_2_.

Conclusions. Activation of the Nrf2 and SIRT1 signaling pathways ameliorated oxidative stress and mitochondrial dysfunction. Pharmacological targeting of Nrf2 signaling molecules may reduce the pathologic changes of aging in the kidney.

## Introduction

Changes with aging represent a major risk factor for the induction of various diseases in organs such as liver, heart, vessels, and kidney [1]. In particular, the kidney ages quickly compared to the other organs and renal age-related change is representatively known to increase in glomerulosclerosis, interstitial fibrosis, arteriosclerosis, and tubular atrophy [2,3]. These changes can occur in various possible biological mechanisms of aging, including expression of senescence genes, changes in hormones, increase in oxidative stress, and damage of mitochondria [4,5]. Oxidative stress has been proposed to promote mitochondrial dysfunction [6]. Mitochondrial biogenesis is not only promoted in association with oxidative stress, but can also be induced in response to aging [7,8].

It is well known that the aging process is associated with the inactivation of the silent information regulator T1 (SIRT1) protein, the activation of the renin–angiotensin system, oxidative stress, and mitochondrial dysfunction [3]. Recently, SIRT1 has been shown to interact with PGC-1α, consequently increasing PGC-1α activity levels, leading to the promotion of mitochondrial biogenesis [9].

The nuclear factor (erythroid-derived 2)-like 2 (Nrf2) protein, a transcription factor that regulates intracellular redox balance and the antioxidants in the cell, regulates inflammation, senescence, and reactive oxygen species (ROS) [10,11]. Upon exposure of cells to oxidative stress, Nrf2 translocates into the nucleus to bind to antioxidant-responsive elements in genes encoding antioxidant enzymes, such as NADPH quinone oxidoreductase (NQO-1), heme oxygenase-1 (HO-1), and superoxide dismutase 1 (SOD1) and 2 (SOD2) [10-12]. Control of the oxidative stress caused by Nrf2 also affects mitochondrial dysfunction [7].

Resveratrol could be useful to treat various diseases; for example, it has anti-cancer, anti-aging, anti-inflammatory effects, such as prolonging lifespan, which have been reported [13]. Resveratrol is known to activate both Nrf2 and SIRT1, and activation of these proteins by resveratrol is regulated by oxidative stress and mitochondrial biogenesis through increased antioxidant protein expression and PGC-1α deacetylation [14].

We hypothesized that the Nrf2 activator resveratrol might ameliorate ageing-related conditions such as renal injury caused by age-related oxidative stress and mitochondrial dysfunction. In this study, we focused on the effects of resveratrol on changes in oxidative stress by Nrf2 signaling and improvement of mitochondria dysfunction through the SIRT1-PGC-1α pathway.

## RESULTS

### Baseline characteristics in aging mice and effects of resveratrol

In the present study, the changes in physical and biochemical characteristics in aging mice before and after treatment of resveratrol were measured. Before treatment of resveratrol, the body weight, food intake, and blood glucose and hemoglobin A1c (HbA1c) levels were not significantly different between the two groups. On the other hand, after treatment with resveratrol, the body weight and kidney weight were lower in the RSV group than those of the control (Cont) group. There was no change in food intake, whereas blood glucose and HbA1c levels were significantly deceased in the RSV group (Table 1).

**Table 1:** Baseline characteristics of control group and RSV group before and after treatment of resveratrol.

**Parameters**	**Before treatment of resveratrol.**		**After treatment of resveratrol.**	
	**Control group**	**RSV group**	**Control group**	**RSV group**
**Body weight (g)**	38.64 ± 1.4	40.72± 1.06	40.24 ± 4.54	34.26 ± 2.18**^†^**
**Right kidney weight (g)**	-	-	0.25 ± 0.01	0.20 ± 0.01*
**Left kidney weight (g)**	-	-	0.26 ± 0.01	0.21 ± 0.01**^†^**
**Food intake (g/day)**	4.62 ± 0.32	4.75 ± 0.24	4.78 ± 0.26	4.72 ± 0.18
**Glucose (mg/dL)**	150 ± 8.43	150 ± 10.43	170 ± 4.6	117.42 ± 8.57**^‡^**
**HbA1c (%)**	4.45 ± 0.08	4.31 ± 0.05	4.55 ± 0.08	4.15 ± 0.06**^†^**

* *P*<0.05, † *P*<0.01, ‡*P*<0.0001 compared with the control group.

### Renal function in aging mice and effects of resveratrol

To investigate renal function, albumin concentration was measured in 24-h urine. With aging, urinary albumin excretion was significantly decreased in the RSV group compared to that in the Cont group (Cont 63.97 ± 8.69 μg/24 h vs. RSV 29.46 ± 5.84 μg/24 h, *P*<0.01). Serum creatinine levels were decreased in the RSV group (Cont 0.67 ± 0.13 mg/dL vs. RSV 0.25 ± 0.02 mg/dL, *P*<0.05), and creatinine clearance was increased in the RSV group compared to that in the Cont group (Cont 0.09 ± 0.01 ml/min vs. RSV 0.26 ± 0.03 ml/min, *P*<0.001). There was no change in blood urea nitrogen (BUN) levels. (Table 2). Previously, we reported that as aging progresses, the 24-h urinary albumin excretion is increased (2-month-old group: 16.5 ± 0.6 μg/24 h; 12-month-old group: 41.8 ± 8.45 μg/24 h; 24-month-old group 65.5 ± 10.45 μg/24 h) [3]. Comparing this result with previous results, resveratrol significantly reduced urinary albumin and serum creatinine, improving creatinine clearance.

**Table 2:** Biochemical characteristics in the aging control group and RSV group.

**Parameters**	**Control group**	**RSV group**
**Albuminuria (μg/24 h)**	63.97 ± 8.69	29.46 ± 5.84**^†^**
**Serum creatinine (mg/dL)**	0.67 ± 0.13	0.25 ± 0.02*
**CrCl (ml/min)**	0.09 ± 0.01	0.26 ± 0.03**^‡^**
**BUN (mg/dL)**	22.14 ± 1.20	25.42 ± 2.42

Control of the albuminuria concentration at 18 months (young) before treatment was 27.26 ± 1.01 μg/24 h.

*P<0.05, †*P*<0.01, ‡*P*<0.001 compared with the control group.

### Renal histology and changes of inflammatory cell infiltration, and apoptosis in aging mice and effects of resveratrol

**Figure 1:**
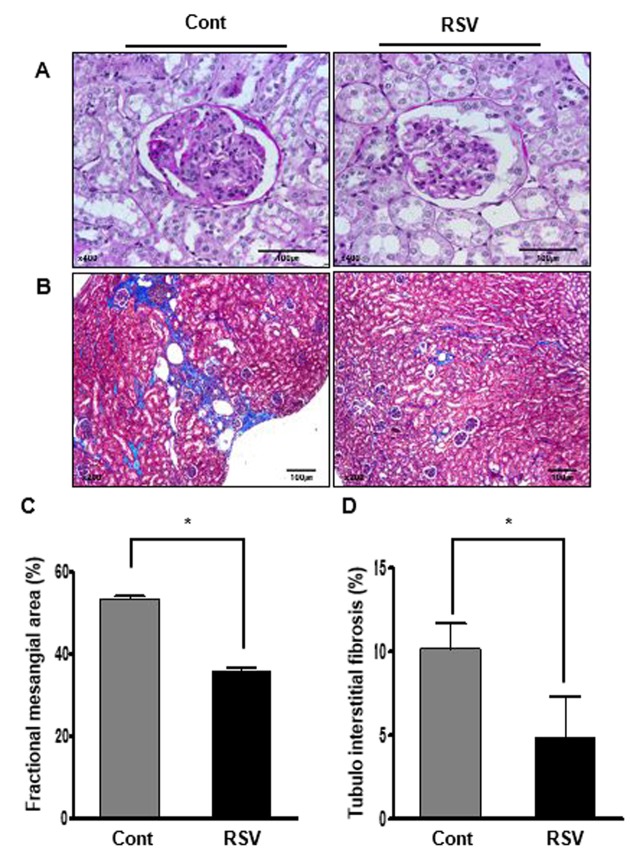
**Effects of resveratrol on aging-related histological renal injury.** Less expansion of the mesangial area (**A**; periodic acid–Schiff (PAS), original magnification ×400) and significantly less tubulointerstitial fibrosis (**B**; Masson’s trichrome, original magnification ×200) were found in the RSV group compared to that in the control (Cont) group. Quantitative assessment of the areas of extracellular matrix in the glomerulus (**C**) and tubulointerstitial fibrosis (**D**) in the control and RSV groups, respectively. (*P<0.001).

**Figure 2:**
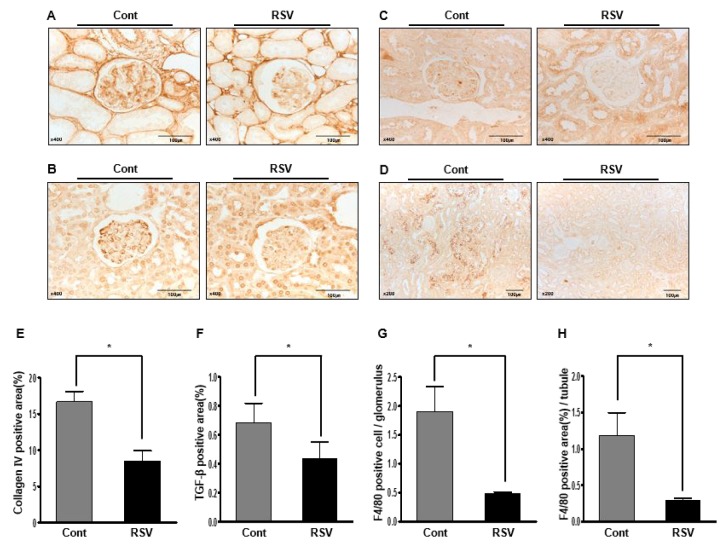
**Effects of resveratrol on the renal phenotypes of collagen IV (Col IV), TGF-β1 and F4/80**. Immunohistochemical staining with Col IV, TGF-β1, and F4/80 in RSV and control (Cont) groups. Representative images of Col IV and TGF-β1 in aging kidney glomerulus (**A**, **B**; original magnification ×400) are shown. In addition, the F4/80-positive cells in glomeruli and F4/80-positive areas in the tubules (**C**, **D**; original magnification ×400) are shown. Expression of Col IV and TGF-β1 was decreased in the RSV group (**E**, **F**). F4/80-positive cells in glomeruli and positive areas in the tubules were observed as significantly smaller in the RSV group (**G**, **H**) (*P<0.001).

**Figure 3:**
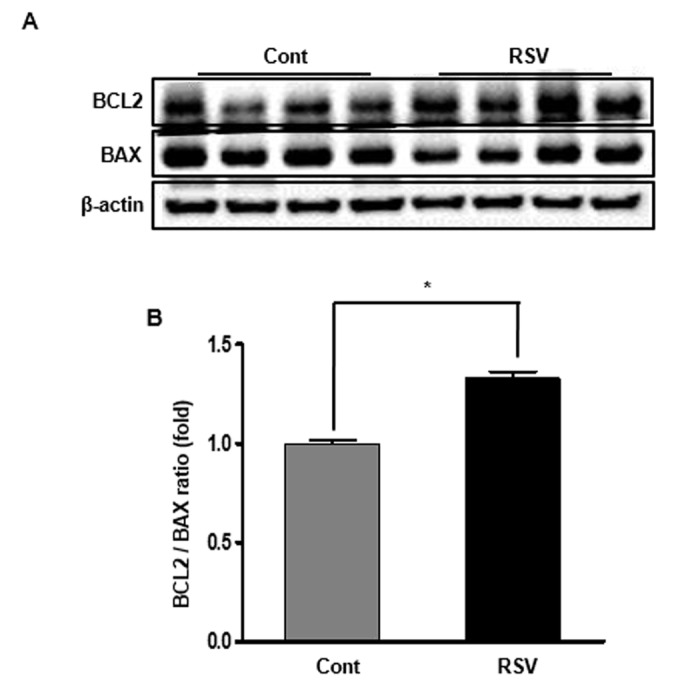
**Effects of resveratrol on the expression of BCL2 and BAX proteins.** Representative western blot analysis of BCL-2 and BAX expression (**A**). BCL-2 protein levels were increased in the RSV group, while BAX protein levels were not different between the two groups (**B**). Quantitative analysis of the results is shown (*P<0.05).

Histological examination showed the fractional mesangial area and tubulointerstitial fibrosis. Mesangial area was smaller in the RSV group than in the Cont group (Cont 53.38 ± 0.72% vs. RSV 35.93 ± 0.63%, *P*<0.001; Figure 1A, C). The area of tubulointerstitial fibrosis was decreased in the RSV group compared to that in the Cont group (Cont 10.17 ± 1.50% vs. RSV 4.83 ± 2.49%, *P*<0.001; Figure 1B, D). The immunohistochemical analysis of collagen IV (Col IV), TGF-β1 and F4/80 is shown in Figure 2. The expression of extracellular matrix Col IV (Cont 16.66 ± 1.47% vs. RSV 8.46 ± 1.45%, *P*<0.001; Figure 2A, E) and the pro-fibrotic growth factor TGF-β1 (Cont 0.68 ± 0.13% vs. RSV 0.43 ± 0.11%, *P*<0.001; Figure 2B, F) were significantly decreased in the RSV group in comparison with the Cont group. F4/80, which is associated with inflammatory cell infiltration, was quantified for positive cells in glomeruli and positive areas in tubules. F4/80-glomerular positive cells (Cont 1.9 ± 0.35 cell vs. RSV 0.48 ± 0.29 cell, *P*<0.001; Figure 2C, G) and tubule-positive areas (Cont 1.18 ± 0.3% vs. RSV 0.29 ± 0.02%, *P*<0.001; Figure 2D, H) in the RSV group were fewer in number than in the Cont group. Changes in renal apoptosis were confirmed by western blot analysis for anti-apoptotic BCL-2 protein and pro-apoptotic BAX protein. There was no difference in the expression of BAX between the two groups, while that of BCL-2 was increased in the RSV group. Consequently, the BCL-2/BAX ratio was significantly higher in the RSV group than in the Cont group (Cont 1 ± 0.01-fold vs. RSV 1.32 ± 0.03-fold, *P*<0.05; Figure 3).

### Renal expression of Nrf2 in aging mice and effects of resveratrol

**Figure 4:**
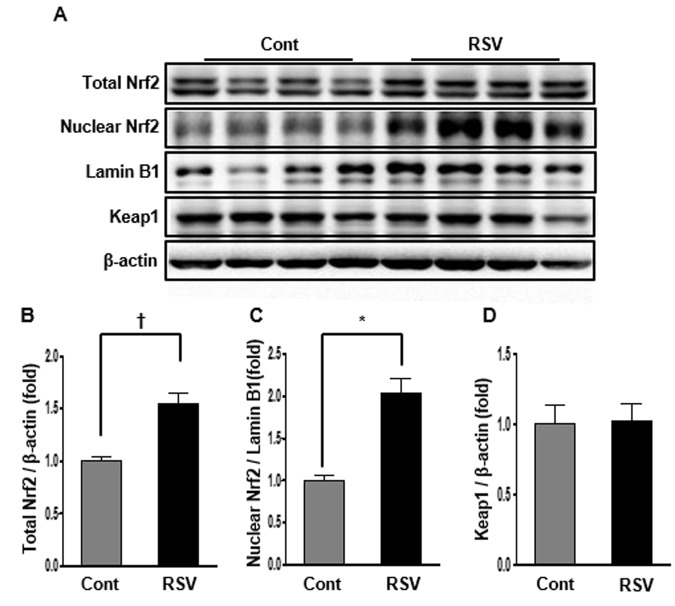
**Effects of resveratrol on the expression of Nrf2-related proteins.** Representative western blot analysis of Nrf2 expression in total and nuclear proteins, and Keap1 expression in total protein (**A**). The results showed that the total and nuclear Nrf2 protein levels were decreased in the RSV group compared to that in the control (Cont) group (**B**, **C**). There were no differences in Keap1 protein expression between both groups (**D**). Quantitative analysis of the results is shown (*P<0.05, **†***P*<0.01).

Nrf2 is a protein that translocates into the nucleus, regulating the expression of antioxidant proteins that protect against oxidative damage by injury and inflammation [10]. Nrf2 expression was confirmed in total and nuclear protein by western blot analysis. Nrf2 levels in total protein were dramatically increased in the RSV group (Cont 1 ± 0.04-fold vs. RSV 1.55 ± 0.09-fold, *P*<0.01; Figure 4A, B). Similarly, Nrf2 protein levels in the nucleus were increased in the RSV group compared to that in the Cont group (Cont 1 ± 0.07-fold vs. RSV 2.02 ± 0.18-fold, *P*<0.05; Figure 4A, C). Keap1, known as a master regulator of the antioxidant response, was not significantly different between the two groups (Cont 1 ± 0.14-fold vs. RSV 1.02 ± 0.12-fold; Figure 4A, D).

### Renal expression of HO-1 and NQO-1 in aging mice and effects of resveratrol

**Figure 5:**
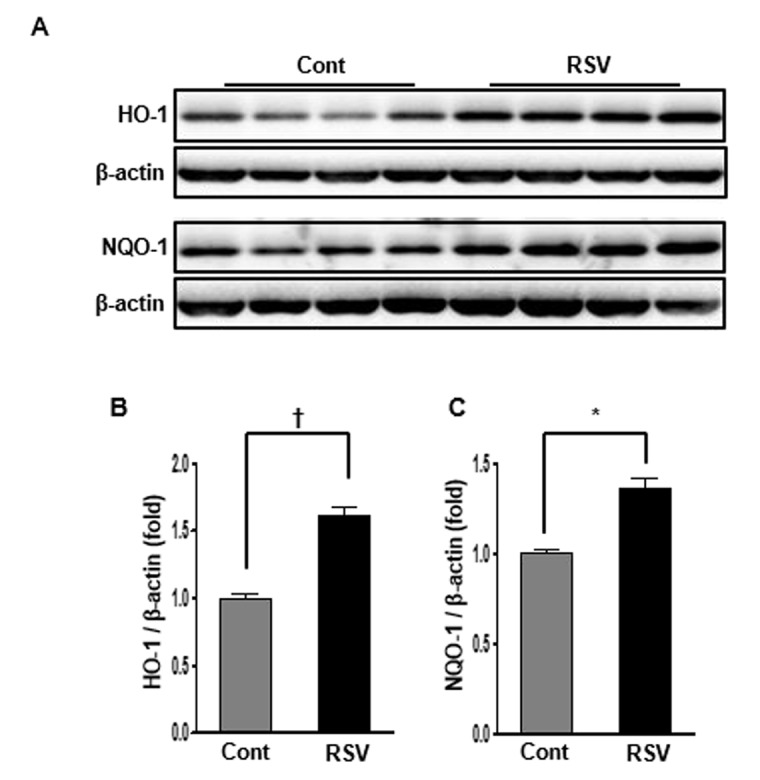
**Effects of resveratrol on the HO-1 and NQO-1 protein expressions.** Representative western blots of HO-1 and NQO-1 protein levels. (**A**) The protein levels of HO-1 and NQO-1 were higher in the RSV group than in the control (Cont) group (**B**, **C**). Quantitative analysis of the results is shown (*P<0.05, †*P*<0.01).

The expression of HO-1, an antioxidant protein, is induced by oxidative stress, and an increase in protein levels is known to protect against oxidative stress. In the present study, HO-1 expression in aging kidney was decreased, and inversely increased in the RSV group (Cont 1 ± 0.03-fold vs. RSV 1.6 ± 0.06-fold, *P*<0.01; Figure 5A, B). NQO-1 is a member of the NAD(P)H dehydrogenase (quinone) family, and this enzyme prevents one of the electron reduction steps of quinones that results in the production of radical species [12]. The expression of NQO-1 was significantly increased in the RSV group compared to that in the Cont group (Cont 1 ± 0.02-fold vs. RSV 1.35 ± 0.06-fold, *P*<0.05; Figure 5A, C).

### Renal expression of SIRT1, phospho-Thr^172^ AMPK and total-AMPK, PPARα, PGC-1α, and ERRα in aging mice and effects of resveratrol

**Figure 6:**
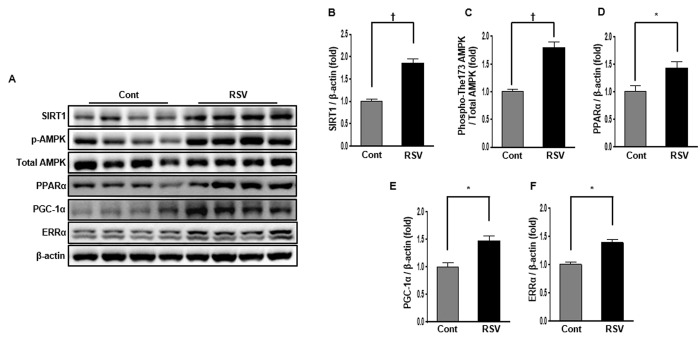
**Effects of resveratrol on the expression of SIRT1-related proteins.** Representative western blots of SIRT1, phospho-Thr^172^ AMPK, PPARα, PGC-1α, and ERR-1α protein levels (**A**). Compared with the control (Cont) group, expression of SIRT1 was significantly increased in the RSV group (**B**). The phospho-Thr^172^ AMPK/total AMPK ratio was also increased in the RSV group (**C**). PPARα, PGC-1α and ERR-1α protein levels were higher in RSV than in Cont (**D**-**F**). Quantitative analysis of the results is shown (*P<0.05, †*P*<0.01).

SIRT1, AMPK, PPARα, PGC-1α, and ERRα protein interactions can regulated mitochondrial biogenesis and reduce mitochondrial ROS produced by cellular oxidative stress [9]. Western blot analysis showed that SIRT1 protein levels were considerably increased in the RSV group compared to those in the Cont group (Cont 1 ± 0.05-fold vs. RSV 1.85 ± 0.10-fold, *P*<0.01; Figure 6A, B). To confirm the changes in the SIRT1 target proteins, we investigated the phospho-Thr^172^/total-AMPK expression ratio, as well as PPARα, PGC-1α, and ERRα expression levels in the aging kidney. The phospho-Thr^172^/total-AMPK protein expression ratio was markedly increased in the RSV group compared to that in the Cont group (Cont 1 ± 0.04-fold vs. RSV 1.79 ± 0.08-fold, *P*<0.01; Figure 6A, C). Also, PPARα levels (Cont 1 ± 0.10-fold vs. RSV 1.42 ± 0.12-fold, *P*<0.05; Figure 6A, D) were statistically increased along with PGC-1α (Cont 1 ± 0.07-fold vs. RSV 1.47 ± 0.08-fold, *P*<0.05; Figure 6A, E) and ERRα expressions (Cont 1 ± 0.05-fold vs. RSV 1.38 ± 0.06-fold, *P*<0.05; Figure 6A, F), which were recovered in the RSV group compared to that in the Cont group.

### Renal expression of SOD1 and SOD2 in aging mice and effects of resveratrol

**Figure 7:**
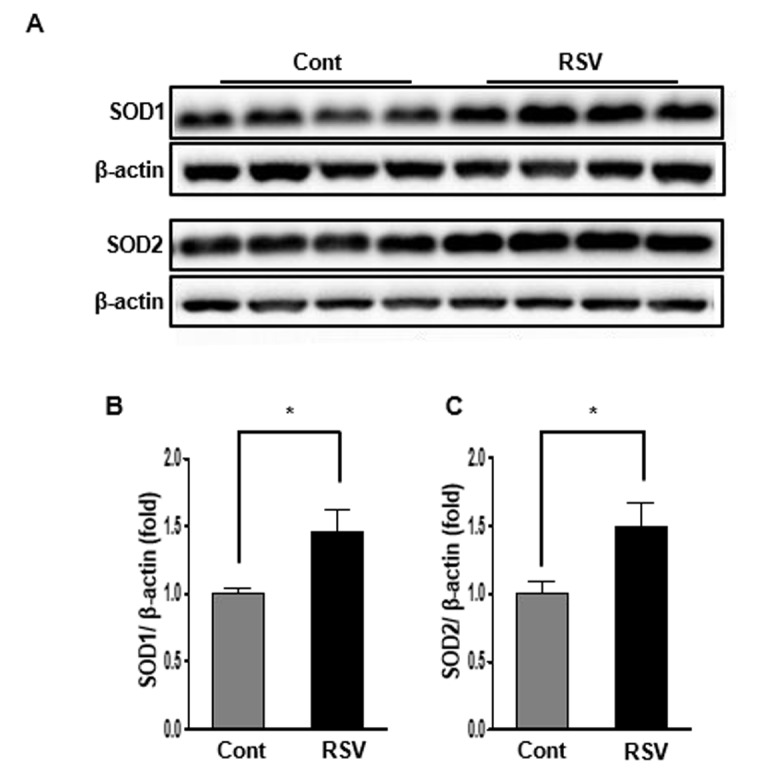
**Effects of resveratrol on the expression of SOD1 and SOD2**. Representative western blots of SOD1 and SOD2 protein levels. (**A**). Expression of SOD1 and SOD2 was significantly increased in the RSV group compared to that in the control (Cont) group (**B**, **C**). Quantitative analysis of the results is shown (*P<0.05).

SOD1 and SOD2 are major antioxidants that are known to have the capacity to limit the detrimental effects of ROS release during oxidative stress. Western blot analysis was used to examine changes in the expression of the antioxidant proteins SOD1 and SOD2. Both expressions of SOD1 and SOD2 were significantly higher in the RSV group than in the Cont group (SOD1: Cont 1 ± 0.04-fold vs. RSV 1.49 ± 0.16-fold, *P*<0.05; Figure 7A, B; SOD2: Cont 1 ± 0.09-fold vs. RSV 1.35 ± 0.18-fold, *P*<0.05; Figure 7A, C).

### Renal cytochrome C oxidase I/IV expression ratio in aging mice and effects of resveratrol

**Figure 8:**
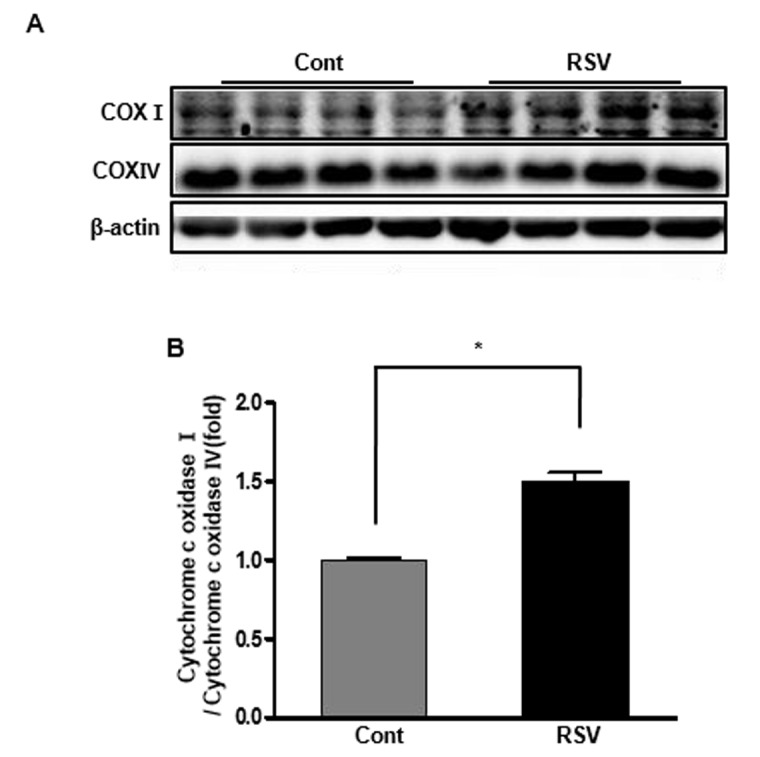
**Effects of resveratrol on the expression of cytochrome c oxidases.** Representative western blots of cytochrome c oxidase I and IV protein levels (**A**). The cytochrome c oxidase I/cytochrome c oxidase IV expression ratio was increased in the RSV group compared to that in the control (Cont) group (**B**). Quantitative analysis of the results is shown (*P<0.05).

Cytochrome c oxidase (as known as complex IV) is an enzyme of the terminal complex in the electron transport chain of mitochondrial oxidative phosphorylation and its subunits I, II, and III are encoded by mitochondrial DNA (mt-DNA) [15,16]. To evaluate its function in the mitochondria, we measured cytochrome c oxidase subunit I (COX I), one of the three subunits encoded by mt-DNA using western blot analysis. Cytochrome C oxidase IV (COX IV) was used as internal control because it remains relatively stable [15]. The COX I/COX IV expression ratio was significantly increased in the RSV group compared to that in the Cont group (Cont 1 ± 0.01-fold vs. RSV 1.5 ± 0.05 fold, *P*<0.05; Figure 8).

### Renal oxidative stress in aging mice and effects of resveratrol

**Figure 9:**
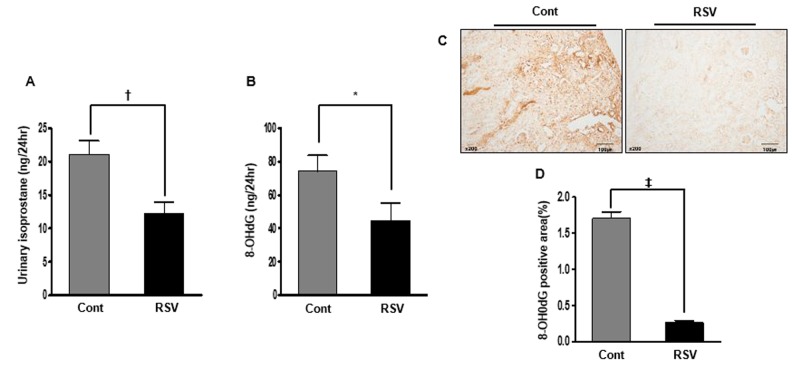
**Effects of resveratrol on renal oxidative stress.** The 24-h urinary 8-epi-prostaglandin F2α (isoprostane) levels were decreased in the RSV group (**A**). Moreover, the 24-h concentration of urinary 8-hydroxy-deoxyguanosine was also decreased in the RSV group compared to that in the control (Cont) group (**B**). Significantly, the positive area expression of 8-OH-dG in renal tissue was decreased in the RSV group (**C**, **D**) (*P<0.05, †*P*<0.01, ‡*P*<0.0001).

To estimate oxidative stress, we measured urinary 8-epi-prostaglandin F2α (isoprostane) and 8-hydroxy-deoxyguanosine (8-OH-dG). As a marker of lipid peroxidation in animals under oxidative stress, urinary isoprostane was significantly decreased in the RSV group (Cont 21.06 ± 2.07 ng/24 h vs. RSV 12.2 ± 1.74 ng/24 h, *P*<0.01; Figure 9A). As another marker oxidative stress, urinary 8-hydroxy-deoxyguanosine is a major product of DNA oxidation. Significantly, the decreased 8-OH-dG concentration in the RSV group was similar to the levels of urinary isoprostane (Cont 74.32 ± 9.50 ng/24 h vs. RSV 44.73 ± 10.48 ng/24 h, *P*<0.05; Figure 9B). Also, the expression of 8-OH-dG by immunohistochemistry in tissues was decreased in the RSV group (Cont 1.70 ± 0.09% vs. RSV 0.26 ± 0.02%, *P*<0.0001; Figure 9C, D).

### Expression of Nrf2 and SIRT1 signaling in the HK2 cells and effects of resveratrol

**Figure 10:**
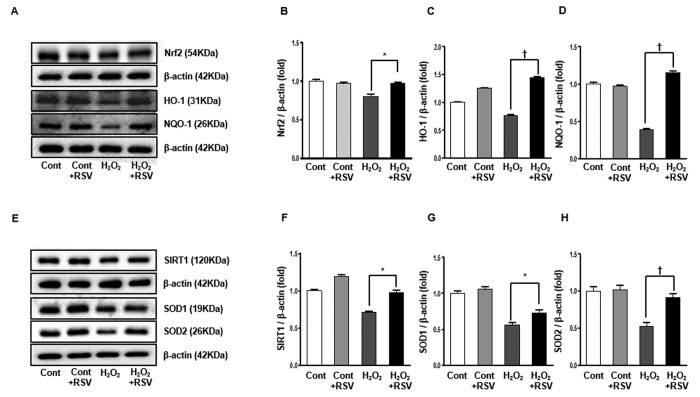
**Effects of resveratrol in HK2 cells.** Representative western blots of Nrf2, HO-1 and NQO-1 protein levels (**A**) and SIRT1, SOD1 and SOD2 protein levels (**E**). The Nrf2 was increased in the RSV group compared to H_2_O_2_ group (**B**). HO-1 as well as NQO-1 were significantly increased in the RSV group (**C**, **D**). Also, the expression of SIRT1 was significantly increased in the RSV group (**F**). Both SOD1 and SOD2 were higher in the RSV group than the H_2_O_2_ group (**G**, **H**). Quantitative analysis of the results is shown (*P<0.05, †*P*<0.001).

We evaluated the effects of resveratrol on H_2_O_2_-induced oxidative stress in cultured HK2 cells. Western blot analyses showed that the expression of Nrf2 was reduced in the H_2_O_2_ group and significantly increased in the RSV group (Cont 1 ± 0.02-fold vs. Cont+RSV 0.97 ± 0.01 fold, H_2_O_2_ 0.80 ± 0.03-fold, H_2_O_2_+RSV 0.97 ± 0.01 fold, *P*<0.05; Figure 10A, B). In addition, expressions of HO-1 and NQO-1, which were significantly decreased in the H_2_O_2_ group, were increased in the RSV group (HO-1: Cont 1 ± 0.01-fold vs. Cont+RSV 1.25 ± 0.007 fold, H_2_O_2_ 0.76 ± 0.009-fold, H_2_O_2_+RSV 1.44 ± 0.01 fold, *P*<0.001; Figure 10A, C; NQO-1: Cont 1 ± 0.02-fold vs. Cont+RSV 0.96 ± 0.01 fold, H_2_O_2_ 0.39 ± 0.01-fold, H_2_O_2_+RSV 1.14 ± 0.03 fold, *P*<0.001; Figure 10A, D). Similarly, the expression of SIRT1 was reduced by exposure to H_2_O_2_ and then recovered after RSV treatment (Cont 1 ± 0.01-fold vs. Cont+RSV 1.18 ± 0.02 fold, H_2_O_2_ 0.70 ± 0.01-fold, H_2_O_2_+RSV 0.97 ± 0.02 fold, *P*<0.05; Figure 10E, F). Both the expressions of SOD1 and SOD2, which are downstream targets of SIRT1, were significantly increased in the RSV group than in the H_2_O_2_ group (SOD1: Cont 1 ± 0.03-fold vs. Cont+RSV 1.05 ± 0.03 fold, H_2_O_2_ 0.56 ± 0.03-fold, H_2_O_2_+RSV 0.72 ± 0.04 fold, *P*<0.001; Figure 10E, G; SOD2: Cont 1 ± 0.06-fold vs. Cont+RSV 1.01 ± 0.06 fold, H_2_O_2_ 0.52 ± 0.05-fold, H_2_O_2_+RSV 0.91 ± 0.04 fold, *P*<0.001; Figure 10E, H).

### Effects of resveratrol on Nrf2 and SIRT1

**Figure 11:**
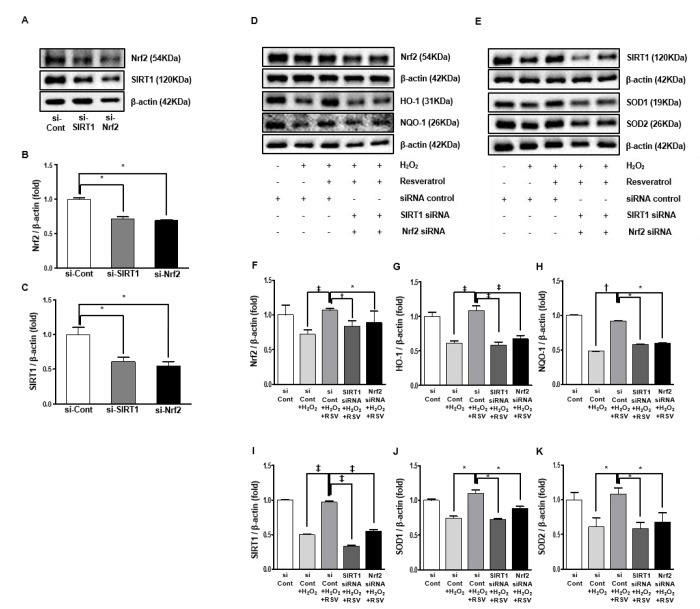
**Small interfering RNA in HK2 cells.** Representative western blots of Nrf2 and SIRT1 protein levels by transfected siRNA for Nrf2 and SIRT1 (**A**). Protein expression of Nrf2 and SIRT1 by transfection with siNrf2 and siSIRT1 were suppressed than the control group (**B**, **C**). Representative western blots of Nrf2, HO-1 and NQO-1 protein levels (**D**) and SIRT1, SOD1 and SOD2 protein levels by transfection with Nrf2 and SIRT1 (**E**). In groups with siNrf2 and siSIRT1 treated with resveratrol in exposure to H_2_O_2_ media, Nrf2, HO-1 and NQO-1 protein expressions were lower compared to the resveratrol group exposed H_2_O_2_ media (**F**-**H**). Similarly, the resveratrol-induced increase in protein levels of SIRT1, SOD1 and SOD2 were inhibited by siNrf2 and siSIRT1 (**J**-**K**). Quantitative analysis of the results is shown (*P<0.05, †*P*<0.01, ‡*P*<0.0001).

We performed additional experiments using small-interfering RNAs (siRNAs) for Nrf2 and SIRT1 in cultured HK2 cells. The expression of Nrf2 was suppressed by transfected siRNA for SIRT1, and that of SIRT1 was suppressed by transfected siRNA for Nrf2. This suggests that Nrf2 and SIRT1 interact with each other (Nrf2: siCont 1 ± 0.01-fold vs. siSIRT1 0.71 ± 0.02 fold, siNrf2 0.69 ± 0.01-fold, *P*<0.001; Figure 11A, B, SIRT1; siCont 1 ± 0.09-fold vs. siSIRT1 0.60 ± 0.06 fold, siNrf2 0.55 ± 0.05-fold, *P*<0.001; Figure 11A, C).

Additional experiment was done to evaluate the whether Nrf2 and SIRT1 was associated with the resveratrol-induced changes. Transfection with Nrf2 and SIRT1 siRNA, respectively, suppressed the resveratrol-induced increase in expressions of Nrf2, HO-1 and NQO-1 (Nrf2; siCont 1 ± 0.13-fold vs. siCont+H_2_O_2_ 0.71 ± 0.06 fold, siCont+H_2_O_2_+RSV 1.06 ± 0.02-fold, siSIRT1+H_2_O_2_+RSV 0.83 ± 0.08 fold, siNrf2+H_2_O_2_+RSV 0.88 ± 0.16 fold, *P*<0.05, *P*<0.001, *P*<0.0001; Figure 11D, F; HO-1: siCont 1 ± 0.05-fold vs. siCont+H_2_O_2_ 0.61 ± 0.03 fold, siCont+H_2_O_2_+RSV 1.08 ± 0.06-fold, siSIRT1+H_2_O_2_+RSV 0.58 ± 0.04 fold, siNrf2+H_2_O_2_+RSV 0.67 ± 0.04 fold, *P*<0.0001; Figure 11D, G; NQO-1: siCont 1 ± 0.009-fold vs. siCont+H_2_O_2_ 0.48 ± 0.003 fold, siCont+H_2_O_2_+RSV 0.91 ± 0.003-fold, siSIRT1+H_2_O_2_+RSV 0.58 ± 0.003 fold, siNrf2+H_2_O_2_+RSV 0.59 ± 0.002 fold, *P*<0.0001; Figure 11D, H). The resveratrol-induced increase in expressions of SIRT1, SOD1 and SOD2 were suppressed by transfection with Nrf2 and SIRT1 siRNA (SIRT1; siCont 1 ± 0.005-fold vs. siCont+ H_2_O_2_ 0.49 ± 0.008 fold, siCont+H_2_O_2_+RSV 0.96 ± 0.02-fold, siSIRT1+H_2_O_2_+RSV 0.33 ± 0.01 fold, siNrf2+H_2_O_2_+RSV 0.54 ± 0.02 fold, *P*<0.0001; Figure 11E, I; SOD1: siCont 1 ± 0.01-fold vs. siCont+H_2_O_2_ 0.74 ± 0.02 fold, siCont+H_2_O_2_+RSV 1.09 ± 0.05-fold, siSIRT1+H_2_O_2_+RSV 0.72 ± 0.01 fold, siNrf2+H_2_O_2_+RSV 0.88 ± 0.02 fold, *P*<0.05; Figure 11E, J; SOD2: siCont 1 ± 0.10-fold vs. siCont+H_2_O_2_ 0.78 ± 0.13 fold, siCont+H_2_O_2_+RSV 1.14 ± 0.08-fold, siSIRT1+H_2_O_2_+RSV 0.80 ± 0.08 fold, siNrf2+H_2_O_2_+RSV 0.83 ± 0.13 fold, *P*<0.05; Figure 11E, K). Altogether, transfection with Nrf2 and SIRT1 siRNA prevented resveratrol-induced anti-oxidative effect in HK2 cells in media treated with H_2_O_2_.

## DISCUSSION

Aging is a multifactorial process characterized by a progressive decline in physiological function. We have already reported the renal changes that occur during aging [3]. In that article, male 2-, 12-, and 24-month-old C57BL/6 mice were compared, and 24-month-old mice displayed increased albuminuria, mesangial volume, and tubulointerstitial fibrosis, and decreased creatinine clearance with aging. Expressions of SOD1 and SOD2 were also decreased in 24-month-old mice. Oxidative stress may be mediated by a decrease in Nrf2, SIRT1, PGC-1α, ERRα, and PPARα expressions [3,17]. In the present study, we investigated whether the Nrf2 activator resveratrol protects against age-related changes in the kidney. Resveratrol-treated aging mice displayed decreased albuminuria and increased creatinine clearance. Age-related renal histological changes such as glomerulosclerosis and tubulointerstitial fibrosis, inflammatory cell infiltration, and apoptosis were attenuated in the resveratrol-treated mice. The expression of SIRT1/AMPK and PPARα was higher in the resveratrol-treated mice than in the control mice. The interaction of Nrf2 with SIRT1/AMPK and PPARα expression was associated with changes in PGC-1α and ERRα signaling pathways. The protein contents of the antioxidant enzymes SOD1, SOD2, HO-1, and NQO1 were higher, and an indicator of mitochondrial dysfunction (COX I/ COX IV ratio) was lower in the resveratrol-treated mice. Inhibiting the SIRT1 pathway with SIRT1-siRNA blocked resveratrol-induced upregulation of Nrf2. Inhibiting the Nrf2 pathway with Nrf2-siRNA also blocked resveratrol-induced upregulation of SIRT1.

Two important issues with aging are mitochondrial dysfunction and oxidative stress [18,19]. Age-related oxidative damage occurs because of an increased rate of generation of oxidants [20] and ROS contribute to the accumulation of oxidative damage of cellular constituents [21,22], The key features of this hypothesis are that increases in oxidants and concomitant failure of antioxidant mechanisms cause structural damage to macromolecules which accumulates with age, leading to a corresponding decline or loss of function [23]. Aged kidneys show increased levels of ROS and thiobarbituric acid-reactive substances, which are associated with lipid oxidative damage [24]. In addition, other markers of oxidative stress and lipid peroxidation, such as isoprostanes, Advanced Glycation End products (AGEs), and increased heme oxygenase, are observed in aged rats [25]. In our previous study in aged mice, urine isoprostane excretion increased with aging, and SOD1 and SOD2 were decreased in aged mice. We observed that oxidative stress may be mediated by a decrease in SIRT1, PGC-1α, ERRα, and PPARα expression [3]. Therefore we suggested that pharmacologically targeting these oxidative stress signaling molecules may reduce the pathologic changes of aging in the kidney.

The appropriate functioning of the Nrf2 pathway is indispensable to counteract oxidative stress and protecting multiple organs and cells [26,27]. Nrf2 gene knockout studies in various organs such as lung, liver, kidney, and brain have shown that dysregulation of Nrf2 severely affects the oxidant/ROS sensitivity and predisposes the system to several pathological changes with aberrant cellular lesions [28]. A deficiency in Nrf2 activity may aggravate oxidative damage and inflammatory injury, which is closely related with oxidative stress and inflammatory-related diseases, including cancer, Alzheimer’s disease, Parkinson's disease, chronic obstructive pulmonary disease, asthma, atherosclerosis, diabetes, multiple sclerosis, osteoarthritis, and rheumatoid arthritis [29-32]. In our previous experiment, Nrf2 protein levels in the nucleus were decreased by the acceleration of the aging process and also showed increases in mesangial volume, tubulointerstitial ﬁbrosis, oxidative stress, and apoptosis in the kidney [17]. The role for Nrf2 in renoprotection has been suggested by various studies. In a model of ischemia–reperfusion injury, renal function, vascular permeability, and survival of Nrf2-knockout mice were significantly worse than those of wild-type mice [33]. Renal damage and interstitial fibrosis by cyclosporin A treatment were relatively higher in Nrf2-knockout mice [34]. The STZ-induced diabetic nephropathy model revealed that Nrf2-null mice developed a severe renal injury with greater oxidative DNA damage than did wild-type mice [35]. In that study, both *in vivo* and *in vitro* systems showed that the Nrf2-mediated protection against diabetic nephropathy is, at least, partially through inhibition of TGF-β1 and reduction of extracellular matrix production. In human renal mesangial cells, high glucose induced ROS production and activated Nrf2 and its downstream genes. Accordingly, pharmacological intervention using Nrf2 activators exerted protective effects against injuries from oxidative stress and inflammation in various *in vitro* and *in vivo* experimental models [36]. In the present study, the Nrf2 activator resveratrol ameliorated dysregulation of Nrf2 signaling. The activation of Nrf2 improved renal function, proteinuria, and pathological changes by decreasing oxidative stress. Because Nrf2 controls a critical cellular defense response against oxidative stress, the activation of Nrf2 is important to maintain the redox balance in aging-related renal injury.

In this study, resveratrol also ameliorated the aging-induced suppression of SIRT1 signaling that may decrease oxidative stress in cooperation with AMPK. This signaling may also aﬀect catabolism, mitochondrial function, angiogenesis, inﬂammation, and insulin resistance [37]. The hypothetical SIRT1 and AMPK cycles regulate each other and share many common target molecules, such as PGC-1α, PPARs, forkhead box (FoxO) proteins, and NF-κB [37]. It has been suggested that the activation of AMPK and SIRT1 allows the concurrent deacetylation and phosphorylation of their target molecules and decreases the susceptibility to aging. Among the seven mammalian sirtuins, SIRT1 and SIRT3 are considered as anti-aging molecules that are expressed in the kidney [38]. It has been shown that Sirt1 activation protects the mouse renal medulla from oxidative injury and yields anti-apoptotic and anti-fibrotic effects in obstructed mouse kidneys [39]. Resveratrol can activate SIRT1, leading to deacetylation of SIRT1 target molecules such as NF-κB and FOXO transcription factors. The inhibition of NF-κB by resveratrol reduces the expression of inflammation mediators. FOXO transcription factors are implicated in the upregulation of antioxidant enzymes and the endothelial-type nitric oxide synthase [40]. A recent study showed that resveratrol plays an important activation role by stabilizing SIRT1/peptide interactions in a substrate-specific manner. This new mechanism highlights the importance of the N-terminal domain in substrate recognition, explains the activity restoration role of resveratrol towards some “loose-binding” substrates of SIRT1, and has significant implications for the rational design of new substrate-specific SIRT1 modulators [41]. In this study, resveratrol also increased SIRT1/AMPK signaling and decreased oxidative stress and mitochondrial dysfunction by regulating PGC-1α and ERRα.

To evaluate the relationship between SIRT1 and Nrf2 pathway, SIRT1–siRNA and Nrf2-siRNA was applied in the presence of resveratrol under H_2_O_2_-treated conditions. Resveratrol significantly reversed H_2_O_2_-induced downregulation of SIRT1 and Nrf2. Inhibiting the SIRT1 pathway with SIRT1-siRNA blocked resveratrol-induced upregulation of Nrf2. Inhibiting the Nrf2 pathway with Nrf2-siRNA also blocked resveratrol-induced upregulation of SIRT1. These data showed SIRT1 and Nrf2 interact in aging process. Recently, Huang K et al also showed the crosstalk between Sirt1 and Nrf2 and the anti-oxidative pathway forms a positive feedback loop to inhibit the protein expressions of fibronectin and TGF-β1 in advanced glycation-end products -treated glomerular mesangial cells [42]. Yoon DS et al found that Nrf2 positively regulates SIRT1 at the mRNA and protein levels via negatively regulating p53 in human mesenchymal stem cells. They showed that blocking the nuclear import of Nrf2 can activate p53, which suppresses SIRT1 promoter activity, resulting in a loss of mesenchymal stem cells stemness [43]. However, the underlying mechanism how Nrf2 regulates SIRT1 levels still needs further investigation.

PPARα is mainly expressed in the liver, intestine, and kidney [44]. PPARα agonists such as fenofibrate bind to PPARα and form a heterodimer complex with the retinoid X receptor. This complex then binds to specific peroxisome proliferator response elements to activate target gene transcription. PPARα activators improve whole body lipid homeostasis by acting primarily in the liver to increase fatty acid degradation and cause overall improvement in lipid homeostasis [45]. Previous studies have demonstrated that the PPARα agonist fenofibrate increases lipolytic enzymes to reduce renal lipid accumulation, and subsequently attenuates inflammation and oxidative stress in a diet-induced obese model [46,47]. More importantly, PPARα agonist also prevents the development of albuminuria and structural disorders [46,47]. In other studies, resveratrol decreased collagen accumulation and fibrosis, urea protein concentration, inflammation, and oxidative stress. These benefits were closely associated with upregulation of lipolytic genes and protein levels in the kidney. This showed that the renoprotective impact of resveratrol may be mediated by shifting the gene expression profile of the renal cells to a state that favors lipolysis [48]. The detailed mechanism by which resveratrol regulates PPARα in renal cells is not well understood. Although a recent study indicated that resveratrol exerted off-target effects on PPAR through its direct interaction with PPARγ and PPARα by bioaffinity chromatography [49] another study showed that resveratrol was able to modulate this binding in a dual fashion [50]. Combined with the present study, resveratrol also modulated the expression of PPARα, which indicated that resveratrol might regulate an upstream target of PPARα. All of these possibilities need to be further explored.

Howitz et al. [51] identified resveratrol as a potent SIRT1 and Nrf2 activator, which boosted the interest in the compound as an energy restriction mimetic [52]. More recently, interest in resveratrol shifted towards its potential to affect metabolic health. In 2006, Lagouge et al. [53] showed that a high dose of resveratrol (400 mg/kg per day) resulted in, among others, improvements in insulin sensitivity, and a reduction in body weight. The latter effect might be dose-dependent, as a lower dose of resveratrol (∼22·5 mg/kg per day) was insufficient to result in weight loss, but it still improved glucose tolerance [54]. The reason for the effect of body weight is not clear, but it has been shown that at higher dose of resveratrol results in an increase in energy expenditure, despite a reduction in voluntary exercise, and this could underlie the effects on body weight [53]. Therefore these anti-metabolic effects also may contribute in aging process. However, the main effect of resveratrol is its strong anti-inflammatory effects. Resveratrol can ameliorate several types of renal injury in animal models, including diabetic nephropathy [48], drug-induced injury [55], ischemia-reperfusion injury [56], sepsis-related injury, and endothelial dysfunction. In addition, Resveratrol can prevent the increase in vasoconstrictors, such as angiotensin II (AII) and endothelin-1 (ET-1), as well as intracellular calcium, in mesangial cells [57]. The mechanism of the protective effects of resveratrol against both acute and chronic kidney injuries is its antioxidant effects and activation of SIRT1 through multiple mechanisms, such as activation of AMPK [58]. A number of recent studies indicate that many of the protective effects of resveratrol could be mediated by SIRT1-independent mechanisms. Among them, the activation of mammalian target of rapamycin (mTOR) signaling pathway is involved in the pathogenesis for several kidney diseases, such as diabetic nephropathy [59].

In conclusion, the Nrf2 activator resveratrol improved renal function, proteinuria, glomerulosclerosis, tubular interstitial fibrosis, inflammation, and apoptosis in this mouse model of age-related renal injury. These protective effects were mediated by the activation of SIRT1/AMPK and PPARα signaling. SIRT1/AMPK and PPARα expression were downregulated with aging and may regulate targets of the Nrf2 signaling system. Recent extensive studies have established the role of Nrf2 signaling in renal protection against oxidative damage, and in the modulation of the inflammatory response. The pharmacological targeting of Nrf2 signaling molecules that share antioxidant and anti-inflammatory efficacies may reduce the pathological changes of aging observed in the kidney.

## MATERIALS AND METHODS

### Animal study

The Animal Care Committee of the Catholic University of Korea approved the experimental protocol. The aged mice were housed in a temperature- and light-controlled environment with a 12:12-h light–dark cycle and had free access to water.

### Animal treatment with resveratrol

Eighteen-month-old male C57BL/6 mice were purchased from the Korea Research Institute of Bioscience and Biotechnology (Chungcheongbuk-do, Republic of Korea). The aged male C57BL/6 mice were divided into two groups: the control group (n=7) received normal mouse chow (PicoLab Rodent Diet 20 5053, LabDiet, St. Louis, MO, USA). The resveratrol-treated group (n=7, RSV) received a mixture of resveratrol (3,5,4'-trihydroxy-trans-stilbene; 40 mg/kg; Sigma, St Louis, MO, USA) and normal chow for 6 months. The concentration of resveratrol and the treatment period were determined by referring to the article of Baur JA et al [60]. The mice were sacrificed at the age of 24 months.

### Urinary and blood measurements

Each mouse was placed in an individual mouse metabolic cage (Tecniplast, Gazzada, Italy) and 24-h urine was collected every 4 weeks. Urine collected at 18 and 24 months was used in the experiment. In aging urine, ELISA kits were used to measure albumin concentration (Albuwell M, Exocell, Philadelphia, PA, USA) and urine creatinine concentration (The Creatinine Companion, Exocell). Blood, serum creatinine, BUN, and HbA1c concentrations of aged mice were measured using i-STAT system cartridges (CHEM8+, Abbott Point of Care Inc., Abbott Park, IL, USA). The fasting blood glucose was measured using an Accu-Chek meter (Roche Diagnostics, St Louis, MO, USA). Creatinine clearance was calculated using the standard formula: [urine creatinine (mg/dL) × urine volume (mL/24 h)] / [serum creatinine (mg/dL) × 1440 (min/24 h)].

### Histology

Fixed kidney tissues in 10% formalin were embedded in low melting point paraffin and cut into 4-μm sections. Kidney tissue sections were processed and stained with periodic acid–Schiff (PAS), and Masson’s trichrome. The fractional mesangial area was quantified in PAS-stained cross-sections. A finding of tubulointerstitial fibrosis was defined as the matrix-rich expansion of the interstitium in Masson’s trichrome. Each of the stains was assessed by measurement at 20 randomly selected positions in seven different sections per animal. All of these sections were examined in a blinded manner using a color image analyzer (TDI Scope Eye, Version 3.5 for Windows, Olympus, Tokyo, Japan), and the data was quantified using ImageJ (Wayne Rasband, US National Institutes of Health).

### Immunohistochemistry

Paraffin sections were deparaffinized in xylene and hydrated in ethanol before staining for markers of fibrosis, inflammation, macrophages and oxidative stress using primary antibodies to Collagen IV (Col IV, Abcam, Cambridge, UK), transforming growth factor-β (TGF-β, R&D Systems, MN, USA), and F4/80 (AbD Serotec, Oxford, UK) and 8-hydroxy-deosyguanosine (8-OH-dG, JaICA, Shizuoka, Japan). Sections were treated with an antigen-unmasking solution consisting of 10 mM sodium citrate, pH 6.0, and were then washed with phosphate-buffered saline (PBS). Sections were incubated with 3% H_2_O_2_ in methanol to block endogenous peroxidase activity. Nonspecific binding was blocked with 10% normal horse serum. After incubation with the primary antibody at 4°C overnight, antibodies were visualized with a peroxidase-conjugated secondary antibody using the Vector Impress kit (Vector Laboratories, Burlingame, CA, USA). Sections were then dehydrated in ethanol, cleared in xylene, and mounted without counterstaining. All sections were assessed using a color image analyzer (TDI Scope Eye, Version 3.5 for Windows) and quantified using ImageJ.

### Western blot analysis

Total proteins from whole kidney tissues and HK2 cells were extracted using a Pro-Prep Protein Extraction Solution (Intron Biotechnology, Gyeonggi-Do, Republic of Korea) according to the manufacturer’s instructions. For Nrf2 expression, nuclear proteins were prepared using the NE-PER nuclear and cytoplasmic extraction kit (Thermo Fisher Scientific, Rockford, IL, USA). Western blot analysis was performed using the following antibodies: Nrf2 (Santa Cruz Biotechnology Inc., Dallas, TX, USA), Keap1 (Santa Cruz Biotechnology Inc.), Lamin B1 (Cell Signaling Technology Inc., Danvers, MA, USA), HO-1 (Cell Signaling Technology Inc.), NQO-1 (Santa Cruz Biotechnology Inc.), SIRT1 (Cell Signaling Technology Inc.), total AMPK (Cell Signaling Technology Inc.), phosphorylated (phospho)-Thr^172^ AMPK (Cell Signaling Technology Inc.), PPARα (Abcam), PGC-1α (Novus Biologicals, Littleton, CO, USA), estrogen-related receptor α (ERRα) (Millipore), SOD1 (Enzo Life Sciences, Farmingdale, NY, USA), SOD2 (Abcam), cytochrome c oxidase I (Santa Cruz Biotechnology) and IV (Cell Signaling Technology Inc.), B-cell leukaemia/lymphoma 2 (BCL-2) (Santa Cruz Biotechnology); BCL-2-associaated X protein (BAX) (Santa Cruz Biotechnology) and β-actin (1:10000, Sigma).

### Renal oxidative stress

To evaluate oxidative stress, we measured the 24-h urinary 8-epi-prostaglandin F2α (isoprostane) and 24-h urinary 8-OH-dG concentrations using ELISA kits (OXIS Health Products Inc., Foster City, CA, USA).

### Cell culture and in vitro study

To investigate the effect of resveratrol on cell damage caused by aging, human renal proximal tubular cells (HK2) purchased from the American Type Culture Collection (ATCC, Manassas, VA, USA) were cultured. HK2 Cells were grown in 100-mm dishes using Keratinocyte-SFM media (GIBCO, Grand Island, NY, USA) with supplement in a humidified atmosphere of 95% air, 5% CO_2_ at 37°C. To perform the experiment, we plated HK2 cells in a 6-well plate for 48hr. When the density of the cells reached 80%, 50µM resveratrol was treated for 24 hr, and H_2_O_2_ (sigma), was exposed at 500 µM for 1 hr to observe the changes [48].

### Small interfering RNA (siRNA) transfection in HK2 cells

Scrambled siRNAs targeting Nrf2 and SIRT1 were purchased from Bioneer (Daejeon, Republic of Korea). We cultured 6-well plates for transfection, and when cells reached 60%, we transfected with lipofectamin 3000 (Invitrogen, Carlsbad, CA, USA) according to the manufacturer’s instructions.

### Statistical analysis

Data are expressed as mean ± standard error (SE). Differences between the groups were examined for statistical significance using ANOVA followed by an unpaired *t-*test as appropriate (SPSS v.19.0, IBM Corp., Armonk, NY, USA). *P*-values <0.05 were considered significant.
